# Supplementary material for: Assessment of Mixed Plasmodium falciparum sera5 Infection in Endemic Burkitt Lymphoma: A Case-Control Study in Malawi
Source: Cancers (Basel). 2021 Apr 2;13(7):1692. doi: 10.3390/cancers13071692 (PMC8038222; doi:10.3390/cancers13071692)
Supplement: Supplementary file 1 [file cancers-13-01692-s001.pdf]

cancers

MDPI

Supplementary Materials: Assessment of Mixed *Plasmodium falciparum sera5* Infection in Endemic Burkitt Lymphoma: A Case-Control Study in Malawi

Nobuko Arisue, George Chagaluka, Nirianne Marie Q. Palacpac, W. Thomas Johnston, Nora Mutalima, Sally Peprah, Kishor Bhatia, Eric Borgstein, George N. Liomba, Steve Kamiza, Nyengo Mkandawire, Collins Mitambo, James J. Goedert, Elizabeth M. Molyneux, Robert Newton, Toshihiro Horii and Sam M. Mbulaiteye [6,*]

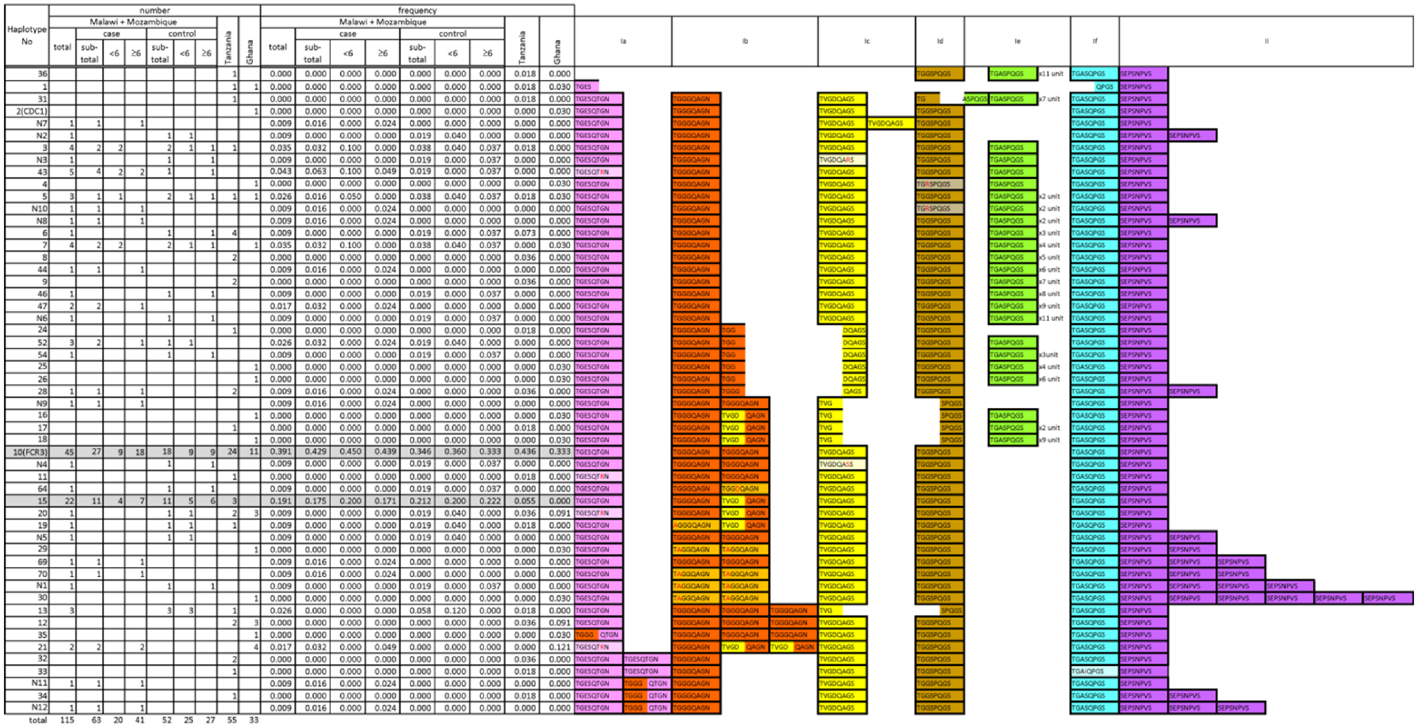

Figure S1. Allele distribution in *Pfsera5* octamer repeat (OctR) region. OctR is classified into two groups; group I at the *N*-terminal consisted of six subgroups (Ia–If) and group II at the C-terminal region. Amino acid substitutions are shown in red. Haplotypes No. 1–70 can be found in *P. falciparum sera5* sequences in database such as NCBI

(https://www.ncbi.nlm.nih.gov/ accessed on 23 October 2020). Haplotypes No. N1 to N12 were newly determined in this study. Haplotypes identical to representative laboratory strains are shown in parentheses. Haplotypes with a frequency greater than 0.2 in the Malawi and Mozambique control group are highlighted in gray. Two of the 63 cases in Malawi and Mozambique were excluded from the grouping by age because the participants ages were unknown.

Haplotype No	number									frequency									sequence
	Malawi + Mozambique							Tanzania	Ghana	Malawi + Mozambique							Tanzania	Ghana	
	total	case			control					total	case			control					
		sub-total	<6	≥6	sub-total	<6	≥6				sub-total	<6	≥6	sub-total	<6	≥6			
1								1	1	0.000	0.000	0.000	0.000	0.000	0.000	0.000	0.018	0.030	TGTVRGDTESISDSSSSS---VNPPANGAGSTPDAKKK
N8	1	1		1						0.009	0.016	0.000	0.024	0.000	0.000	0.000	0.000	0.000	TGTVRGDTESISDSSSSSSSSS-------------------------------------VNPPANGAGSTPDAKKK
2	1	1	1							0.009	0.016	0.050	0.000	0.000	0.000	0.000	0.000	0.000	TGTVRGDTESISDSSSSSSSSSSS-----------------------------------VNPPANGAGSTPDAKKK
3	7	3	1	2	4	3	1	2	1	0.061	0.048	0.050	0.049	0.077	0.120	0.037	0.036	0.030	TGTVRGDTESISDSSSSSSSSSSSSSSS-------------------------------VNPPANGAGSTPDAKKK
69	1	1		1						0.009	0.016	0.000	0.024	0.000	0.000	0.000	0.000	0.000	TGTVRGDTESISDSSSSSSSSSSSSSSSSSSS---------------------------VNPPANGAGSTPDAKKK
5	17	10	2	8	7	2	5	6	5	0.148	0.159	0.100	0.195	0.135	0.080	0.185	0.109	0.152	TGTVRGDTESISDSSSSSSSSSSSSSSSSSSSSS-------------------------VNPPANGAGSTPDAKKK
6								1		0.000	0.000	0.000	0.000	0.000	0.000	0.000	0.018	0.000	TGTVRGDTEPISDSISSSSSSSSSSSSSSSSSSS-------------------------VNPPANGAGSTPDAKKK
7(K1)	2	2		2					2	0.017	0.032	0.000	0.049	0.000	0.000	0.000	0.000	0.061	TGTVRGDTEPISDSSSSSSSSSSSSSSSSSSSSS-------------------------VNPPANGAGSTPDAKKK
N1	1				1		1			0.009	0.000	0.000	0.000	0.019	0.000	0.037	0.000	0.000	TGTVRGDTESISDSSSSSSSSSSSSSSSSSSSSS-------------------------INPPANGAGSTPDAKKK
8	4	3		3	1	1		3	1	0.035	0.048	0.000	0.073	0.019	0.040	0.000	0.055	0.030	TGTVRGDTESISDSSSSSSSSSSSSSSSSSSSSSSS-----------------------VNPPANGAGSTPDAKKK
9	1	1	1					2	2	0.009	0.016	0.050	0.000	0.000	0.000	0.000	0.036	0.061	TGTVRGDTEPISDSSSSSSSSSSSSSSSSSSSSSSS-----------------------VNPPANGAGSTPDAKKK
N9	1	1		1						0.009	0.016	0.000	0.024	0.000	0.000	0.000	0.000	0.000	TGTVRGDTEPISDSSSSSSSSSSSSSSSSSSSSSSS-----------------------VNPPANGAGYTPDAKKK
10	1				1	1		2	2	0.009	0.000	0.000	0.000	0.019	0.040	0.000	0.036	0.061	TGTVRGDTESISDSSSSSSSSSSSSSSSSSSSSSSSSS---------------------VNPPANGAGSTPDAKKK
11	1				1	1		2		0.009	0.000	0.000	0.000	0.019	0.040	0.000	0.036	0.000	TGTVRGDTESISDSSSSSSSSSSSSSSSSSSSSSSSSSSS-------------------VNPPANGAGSTPDAKKK
12									1	0.000	0.000	0.000	0.000	0.000	0.000	0.000	0.000	0.030	TGTVRGDTESISDSSSSSSSSSSSSSSSSSSSSSSSSSSSSS-----------------VNPPANGAGSTPDAKKK
13	1	1		1				2	1	0.009	0.016	0.000	0.024	0.000	0.000	0.000	0.036	0.030	TGTVRGDTESISDSSSSSSSSSSSSSSSSSSSSSSSSSSSSSSS---------------VNPPANGAGSTPDAKKK
14	1				1		1		1	0.009	0.000	0.000	0.000	0.019	0.000	0.037	0.000	0.030	TGTVRGDTEPISDSSSSSSSSSSSSSSSSSSSSSSSSSSSSSSS---------------VNPPANGAGSTPDAKKK
15									1	0.000	0.000	0.000	0.000	0.000	0.000	0.000	0.000	0.030	TGTVRGDTESISDSRSSSSSSSSSSSSSSSSSSSSSSSSSSSSSSS-------------VNPPANGAGSTPDAKKK
16	2	1	1		1		1		1	0.017	0.016	0.050	0.000	0.019	0.000	0.037	0.000	0.030	TGTVRGDTESISDSSSSSSSSSSSSSSSSSSSSSSSSSSSSSSSSS-------------VNPPANGAGSTPDAKKK
17									1	0.000	0.000	0.000	0.000	0.000	0.000	0.000	0.000	0.030	TGTVRGDTESISDSSSSSSSSSSSSSSSSSSSSSSSSSSSSSSSSSSSSSSSSSSSS----VNPPANGAGSTPDAKKK
18									1	0.000	0.000	0.000	0.000	0.000	0.000	0.000	0.000	0.030	TGTVRGDTESISDSSSSSSSSSSSSSSSSSSSSSSSSSSSSSSSSSSSSSSSSSSSSSS--VNPPANGAGSTPDAKKK
19								1		0.000	0.000	0.000	0.000	0.000	0.000	0.000	0.018	0.000	TGTVRGDTESISDSSSSSSSSSSSSSSS-------------------------------VNPPANGAGSTPDAKKR
N10	1	1	1							0.009	0.016	0.050	0.000	0.000	0.000	0.000	0.000	0.000	TGTVRGDTEPISDSSSSSSSSSSSSSSS-------------------------------VNPPANGAGSTPDAKKR
20	6	2		2	4	2	2	1	1	0.052	0.032	0.000	0.049	0.077	0.080	0.074	0.018	0.030	TGTVRGDTESISDSSSSSSSSSSSSSSSSSSSSS-------------------------VNPPANGAGSTPDAKKR
21	7	4		3	3		3	3		0.061	0.063	0.000	0.073	0.058	0.000	0.111	0.055	0.000	TGTVRGDTEPISDSSSSSSSSSSSSSSSSSSSSS-------------------------VNPPANGAGSTPDAKKR
22								1		0.000	0.000	0.000	0.000	0.000	0.000	0.000	0.018	0.000	TGTVRGDTEPISDSSSSSSSSSSSSSSSSSSSSSSSSS---------------------VNPPANGAGSTPDAKKR
N6	1	1		1						0.009	0.016	0.000	0.024	0.000	0.000	0.000	0.000	0.000	TGKVRGDTEPISDSSSSSSSSSSSSSSSSSSSSSSSSS---------------------VNPPANGAGSTPDAKKR
N3	1				1	1				0.009	0.000	0.000	0.000	0.019	0.040	0.000	0.000	0.000	TGTVRGDTEPISDSSSSSSSSSSSSSSSSSSSSSSSSSSSSS-----------------VNPPANGAGSTPDAKKR
23	5	2	1	1	3	1	2	2		0.043	0.032	0.050	0.024	0.058	0.040	0.074	0.036	0.000	TGTVRGDTESISDSSSSSSSSSSSSSSSSSSSSSSSSSSSSSSS---------------VNPPANGAGSTPDAKKR
24	17	12	7	4	5	3	2	1		0.148	0.190	0.350	0.098	0.096	0.120	0.074	0.018	0.000	TGTVRGDTEPISDSSSSSSSSSSSSSSSSSSSSSSSSSSSSSSS---------------VNPPANGAGSTPDAKKR
25								1		0.000	0.000	0.000	0.000	0.000	0.000	0.000	0.018	0.000	TGTVRGDTEPISDSSSSSSSSSSS-----------------------------------VNPPANGPDSPTVKPPR
78	2	1	1		1		1			0.017	0.016	0.050	0.000	0.019	0.000	0.037	0.000	0.000	TGTVRGDTESISDSSSSSSSSSSSSSSSSSSSSS-------------------------VNPPANGPDSPTVKPPR
26	1	1		1				4		0.009	0.016	0.000	0.024	0.000	0.000	0.000	0.073	0.000	TGTVRGDTEPISDSSSSSSSSSSSSSSSSSSSSS-------------------------VNPPANGPDSPTVKPPR
79	1				1		1			0.009	0.000	0.000	0.000	0.019	0.000	0.037	0.000	0.000	TGTVRGDTESISDSSSSSSSSSSSSSSSSSSSSSSSSS---------------------VNPPANGPDSPTVKPPR
27								1		0.000	0.000	0.000	0.000	0.000	0.000	0.000	0.018	0.000	TGTVRGDTEPISDSSSSSSSSSSSSSSSSSSSSSSSSSSSSS-----------------VNPPANGPDSPTVKPPR
28	14	6	3	3	8	4	4	5		0.122	0.095	0.150	0.073	0.154	0.160	0.148	0.091	0.000	TGTVRGDTESISDSSSSSSSSSSSSSSSSSSSSSSSSSSSSSSS---------------VNPPANGPDSPTVKPPR
29									1	0.000	0.000	0.000	0.000	0.000	0.000	0.000	0.000	0.030	TGTVRGDTEPISDSSSSSSSSSSSSSSSSSSSSSSSSSSSSSSS---------------VNPPANGPDSPTVKPPR
N2	2				2	1	1			0.017	0.000	0.000	0.000	0.038	0.040	0.037	0.000	0.000	TGTVRGDTESISDSSSSSSSSSSSSSSSSSSSSSSSSSSSSSSSSSSS-----------VNPPANGPDSPTVKPPR
80										0.000	0.000	0.000	0.000	0.000	0.000	0.000	0.000	0.000	TGTVRGDTESISDSSSSSSSSSSSSSSSSSSSSSSSSSSSSSSSSSSSSSSSS------VNPPANGPDSPTVKPPR
30	1				1	1		3	1	0.009	0.000	0.000	0.000	0.019	0.040	0.000	0.055	0.030	TGTVRGDTEPISDSSSSSSSSSSSSSSS-------------------------------ESLPANGPDSPTVKPPR
32								1		0.000	0.000	0.000	0.000	0.000	0.000	0.000	0.018	0.000	TGTVRGDTESISDSSSSSSSSSSSSSSSSSSS---------------------------ESLPANGPDSPTVKPPR
33								1		0.000	0.000	0.000	0.000	0.000	0.000	0.000	0.018	0.000	TGTVRGDTEPISDSSSSSSSSSSSSSSSSSSSSS-------------------------ESLPANGPDSPTVKPPR
34	4	2	1	1	2	1	1	1		0.035	0.032	0.050	0.024	0.038	0.040	0.037	0.018	0.000	TGTVRGDTESISDSSSSSSSSSSSSSSSSSSSSSSS-----------------------ESLPANGPDSPTVKPPR
84	1				1	1				0.009	0.000	0.000	0.000	0.019	0.040	0.000	0.000	0.000	TGTVRGDTEPISDSSSSSSSSSSSSSSSSSSSSSSS-----------------------ESLPANGPDSPTVKPPR
35	1	1		1				1		0.009	0.016	0.000	0.024	0.000	0.000	0.000	0.018	0.000	TGTVRGDTESISDSSSSSSSSSSSSSSSSSSSSSSSSS---------------------ESLPANGPDSPTVKPPR
85	1				1	1				0.009	0.000	0.000	0.000	0.019	0.040	0.000	0.000	0.000	TGTVRGDTEPISDSSSSSSSSSSSSSSSSSSSSSSSSS---------------------ESLPANGPDSPTVKPPR
36(3D7)									1	0.000	0.000	0.000	0.000	0.000	0.000	0.000	0.000	0.030	TGTVRGDTEPISDSSSSSSSSSSSSSSSSSSSSSSSSSSS-------------------ESLPANGPDSPTVKPPR
37								2		0.000	0.000	0.000	0.000	0.000	0.000	0.000	0.036	0.000	TGTVRGDTESISDSSSSSSSSSSSSSSSSSSSSSSSSSSS-------------------ESLPANGPDSPTVKPPR
N5	1	1		1						0.009	0.016	0.000	0.024	0.000	0.000	0.000	0.000	0.000	TGTVRGDTEPISDSSSSSSSSSSSSSSSSSSSSSSSSSSS-------------------ESLPANGPDSPPVKPPR
38									3	0.000	0.000	0.000	0.000	0.000	0.000	0.000	0.000	0.091	TGTVRGDTEPISDSSSSSSSSSSSSSSSSSSSSSSSSSSSSS-----------------ESLPANGPDSPTVKPPR
39								1		0.000	0.000	0.000	0.000	0.000	0.000	0.000	0.018	0.000	TGTVRGDTESISDSSSSSSSISSSSSSSSSSSSSSSSSSSSS-----------------ESLPANGPDSPTVKPPR
40	1	1		1				1		0.009	0.016	0.000	0.024	0.000	0.000	0.000	0.018	0.000	TGTVRGDTESISDSSSSSSSSSSSSSSSSSSSSSSSSSSSSS-----------------ESLPANGPDSPTVKPPR
42	1	1		1					2	0.009	0.016	0.000	0.024	0.000	0.000	0.000	0.000	0.061	TGTVRGDTEPISDSSSSSSSSSSSSSSSSSSSSSSSSSSSSSSS---------------ESLPANGPDSPTVKPPR
43	1				1	1		1		0.009	0.000	0.000	0.000	0.019	0.040	0.000	0.018	0.000	TGTVRGDTESISDSSSSSSSSSSSSSSSSSSSSSSSSSSSSSSS---------------ESLPANGPDSPTVKPPR
46	1	1		1				1	1	0.009	0.016	0.000	0.024	0.000	0.000	0.000	0.018	0.030	TGTVRGDTEPISDSSSSSSSSSSSSSSSSSSSSSSSSSSSSSSSSS-------------ESLPANGPDSPTVKPPR
49									1	0.000	0.000	0.000	0.000	0.000	0.000	0.000	0.000	0.030	TGTVRGDTEPISDSSSSSSSSSSSSSSSSSSSSSSSSSSSSSSSSSSSS---------ESLPANGPDSPTVKPPR
50									1	0.000	0.000	0.000	0.000	0.000	0.000	0.000	0.000	0.030	TGTVRGDTESISDSSSSSSSISSSSSSSSSSSSSSSSSSSSSSSSSSSSS---------ESLPANGPDSPTVKPPR
N4	1				1		1			0.009	0.000	0.000	0.000	0.019	0.000	0.037	0.000	0.000	TGTVRGDTESISDSSSSSSSSSSSSSSSSSSSSSSSSSSSSSSSSSSSSSSSSSSSS----ESLPANGPDSPTVKPPR
54								1		0.000	0.000	0.000	0.000	0.000	0.000	0.000	0.018	0.000	TGTVRGDTEPISDSSSSSSSSSSSSSSSSSSSSSSSSSSSSSSSSSSSSSSSSSSSSSS--ESLPANGPDSLTVKPPR
N7	1	1		1						0.009	0.016	0.000	0.024	0.000	0.000	0.000	0.000	0.000	TGTVRGDTEPISDSSSSSSSSSSSSSSSSSSSSSSSSSSSSSSSSSSSSSSSSSSSSSSSSESLPANGPDSPTVKPPR
total	115	63	20	41	52	25	27	55	33										

Figure S2. Allele distribution in SERA5 serine repeat (SerR) region. No. 1–85 can be found in *P. falciparum sera5* sequences available in database such as NCBI (https://www.ncbi.nlm.nih.gov/ accessed on 23 October 2020). Haplotypes No. N1 to N10 were newly determined in this study. Haplotypes identical to representative laboratory strains are shown in parentheses. Amino acid variations are shown in colour. Haplotypes with a frequency greater than 0.1 in the Malawi and Mozambique

control group are highlighted in gray. Two of the 63 cases in Malawi and Mozambique were excluded from the grouping by age because the participants ages were unknown.

Table S1. Primers for PCR amplification and sequencing.

**Serine Repeat Antigen 5 Gene (sera5)**		**5′ ==> 3′**
Primers used for PCR amplification		
3.3 kb fragment		
1st PCR	sera5-5F0	GATAATCCGAAATCTTAAAATGTTACAAA
	sera5-3R0	GAGGTACATGATAAATTAAAGATATTATACTACCTTAATAA
2nd PCR	sera5-5F3	TTACGCATACACAAACATTTGTCATTA
	sera5-3R2	CTACCTTAATAAAATGAATAATGGAGAGTTATGCCCTATT
5′ -half fragment		
1st PCR	sera5-5F0	GATAATCCGAAATCTTAAAATGTTACAAA
	sera5-R0	CATCCCATAATTATCTAAGGTACCTGTTGTA
2nd PCR	sera5-5F3	TTACGCATACACAAACATTTGTCATTA
	sera5-R2	GTTGTATCTACGTCTTTAAGTAAACTACAGTAA
3′ -half fragment		
1st PCR	sera5-F1	CAAATACCTATCTGAAGATATTGTAAGTAACTTCA
	sera5-3R0	GAGGTACATGATAAATTAAAGATATTATACTACCTTAATAA
2nd PCR	sera5-F2	GCTGAGACAGAAGATGATGATGAAGATGATTATACT
	sera5-3R2	CTACCTTAATAAAATGAATAATGGAGAGTTATGCCCTATT
Primers used for sequencing		
3.3 kb fragment	sera5-FA	GTTATAAAATGTACAGGAGAAAGTCAAACA
	sera5-FB	GTAAAACTACCATCAAATGGTACAA
	sera5-FC	GATAACAAAGTTGATGTAAGAAAGTATTT
	sera5-FD	GAAAAATGTGATACCTTAGCTTCCA
	sera5-FE	CTTTATCATATGATAACTCAGA
	sera5-FF	GTATGGACCAACTCATTGTCA
	sera5-RA	ATACAGCTGCATTACGGAAT
	sera5-RB	GTTATTTACCTGGAATGTCTGA
	sera5-RC	ATACTTTCAGTAGTATCTTTTGTT
	sera5-RD	CGTTACATGGACCAGTAACTT
	sera5-RE	TCATCACCACATAAGTTCTG
	sera5-RF	CGTTATTTGTATCCAATTTGGA
5′ -half fragment	sera5-FA	GTTATAAAATGTACAGGAGAAAGTCAAACA
	sera5-FB	GTAAAACTACCATCAAATGGTACAA
	sera5-FC	GATAACAAAGTTGATGTAAGAAAGTATTT
	sera5-FD	GAAAAATGTGATACCTTAGCTTCCA
	sera5-R2	GTTGTATCTACGTCTTTAAGTAAACTACAGTAA
	sera5-RB	GTTATTTACCTGGAATGTCTGA
	sera5-RC	ATACTTTCAGTAGTATCTTTTGTT
	sera5-RD	CGTTACATGGACCAGTAACTT

3′ -half fragment	sera5-F2	GCTGAGACAGAAGATGATGATGAAGATGATTATACT
	sera5-FE	CTTTATCATATGATAACTCAGA
	sera5-FF	GTATGGACCAACTCATTGTCA
	sera5-RE	TCATCACCACATAAGTTCTG
	sera5-RF	CGTTATTTGTATCCAATTTGGA

Table S2. Odds ratio (OR) and 95% confidence interval (CI) for eBL case status in univariate and multivariate logistic regression models.

Characteristics with	Univariate All subjects	Bivariate association with Sera5 Sequence	Sera5 Sequence Adjusted*	Sera5 PCR Adjusted‡
		OR (95% CI)		
Sera 5 PCR 2				
Negative	*Ref*	--†		*Ref*
Positive	1.17 (0.76, 1.81)			1.15 (0.68, 1.93)
Sera5 sequence				
Single	*Ref*	--†	*Ref*	
Mixed	2.18 (1.12, 4.26)		2.40 (1.11, 5.17)	
Diversity score				
<Mean	*Ref*	*Ref*	*Ref*	*Ref*
≥Mean	1.25 (0.69, 2.29)	0.95 (0.49, 1.82)	0.66 (0.32, 1.39)	0.85 (0.46, 1.56)
Sera5 sequence				
Single		*Ref*		
Mixed		2.23 (1.10, 4.53)		
Log Pf DNA copy number				
<Mean	*Ref*	*Ref*	*Ref*	*Ref*
≥Mean	1.74 (0.95, 3.20)	1.38 (0.72, 2.64)	1.66 (0.79, 3.49)	2.11 (1.10, 4.01)
Sera5 sequence				
Single		*Ref*		
Mixed		1.95 (0.96, 3.95)		
Proportion of SNPs called				
<Mean	*Ref*	*Ref*	--†	--†
≥Mean	1.09 (0.55, 2.19)	0.89 (0.43, 1.84)		
Sera5 sequence				
Single		*Ref*		
Mixed		2.22 (1.13, 4.38)		
Gender				
Female	*Ref*	*Ref*	*Ref*	*Ref*
Male	1.09 (0.59, 2.01)	1.01 (0.54, 1.88)	0.81 (0.41, 1.57)	0.99 (0.62, 1.58)
Sera5 sequence				
Single		*Ref*		
Mixed		2.18 (1.12, 4.26)		
Age group				
0–5 yrs	*Ref*	*Ref*	*Ref*	*Ref*
6–10 yrs	2.27 (1.15, 4.52)	2.76 (1.35, 5.66)	2.96 (1.42, 6.17)	2.79 (1.66, 4.68)
11–15 yrs	0.62 (0.24, 1.58)	0.71 (0.27, 1.86)	0.67 (0.25, 1.78)	0.66 (0.35, 1.26)
Missing	2.17 (0.40, 11.94)	1.96 (0.34, 11.20)	2.18 (0.37, 12.89)	1.57 (0.48, 5.11)
Sera5 sequence				
Single		*Ref*		
Mixed		2.44 (1.12, 4.26)		

* Sera5 Seq Adjusted for diversity score, DNA copy number, gender and age. ‡ Sera5 PCR Adjusted for diversity score, DNA copy number, gender and age. † Analysis for this variable omitted in the analysis.
